# Life’s essential 8 and risk of progression to diabetes among young adults with prediabetes

**DOI:** 10.1038/s41598-025-19472-y

**Published:** 2025-10-13

**Authors:** Dragana Lovre, Yuanhao Zu, Hei Yuen Cheung, Yilin Yoshida

**Affiliations:** 1 Medicine Service, Section of Endocrinology, Southeast Louisiana Veterans Health Care System, New Orleans, LA 70119 USA; 2 Department of Medicine, Section of Endocrinology and Metabolism, Tulane University School of Medicine, 1430 Tulane Ave., New Orleans, LA 70112 USA; 3 Tulane Center of Excellence in Sex-Based Precision Medicine, New Orleans, LA 70112 USA; 4 Department of Statistics and Data Sciences, School of Public Health and Tropical Medicine, Tulane University, New Orleans, LA 70112 USA; 5 Tulane University School of Medicine, New Orleans, LA 70112 USA

**Keywords:** Prediabetes, Type 2 diabetes, Young adults, Cardiovascular disease risk, Lifestyle intervention, Lifestyle habits, Preventive medicine, Type 2 diabetes, Pre-diabetes

## Abstract

The aim of the study was to assess Life’s Essential 8 (LE8) trajectories by glycemic status change and examine their association with diabetes progression risk in young adults with prediabetes (preDM). We used the Coronary Artery Risk Development in Young Adults study (CARDIA) Year 7–30 data to estimate trends of LE8 scores among preDM participants (impaired fasting glucose, impaired oral glucose tolerance, and/or HbA1c 5.7–6.4%) by status of progression, consistent preDM, and regression. We used logistic regression to evaluate the progression risk with LE8. Among 974 preDM participants, 34% progressed to diabetes, 28% remained preDM, and 38% regressed to euglycemia. Over a mean follow-up of 13 years, the progression group had the greatest decline in LE8 scores. Ideal LE8 scores (vs. moderate or poor) were associated with a 40–90% reduction in the progression risk. In conclusion, maintaining optimal cardiovascular health may prevent diabetes progression among young adults with preDM.

**Supplementary Information:**

The online version contains supplementary material available at 10.1038/s41598-025-19472-y.

## Introduction

The rising incidence of diabetes in young adults is linked with an increased risk of premature cardiovascular disease (CVD)^1,2^. Among young adults with prediabetes (preDM), the extent to which modifiable risk factors mitigate the risk of diabetes progression remains unclear. The American Heart Association (AHA)’s Life’s Essential 8 provides a numeric framework based on eight key modifiable risk factors (including diet, physical activity (PA), nicotine exposure, sleep health, body mass index (BMI), blood lipids, blood glucose, and blood pressure (BP)^3^. Each component is scored 0–100, creating a composite cardiovascular health (CVH) score to help providers and scientists evaluate and monitor cardiovascular health over time^3^. Higher LE8 scores (better CVH) have been associated with a lower risk of CVD, mortality, and numerous other health outcomes in the general population^4^. However, data is lacking regarding the CVH trends, disparities, and association with disease progression in young adults living with preDM. In this study, we used the Coronary Artery Risk Development in Young Adults study (CARDIA) Year 7–30 data to estimate trends of LE8 scores among participants. We aimed to examine trajectories of the LE8 scores, in young adults with preDM by glycemic status changes overall, and by sex and race. We also evaluated the relationship between LE8 scores and progression risk, hypothesizing that maintaining ideal or moderate CVH metrics would reduce the risk of diabetes.

## Results

Our study included data from 3026 young adults, with 974 preDM participants (43 ± 7 years, 39% women) and 2052 consistent euglycemic participants (32 ± 4 years, 63% women) (Table 1 and Supplemental Fig. 1). Over a mean follow-up of 13 ± 7 years among participants identified as preDM via criteria of impaired fasting glucose (IFG) individuals, 34% progressed to diabetes (39 ± 7 years, 39% women), 28% remained in a consistent preDM state (46 ± 5 years, 40% women), 38% regressed to normal glucose levels (44 ± 8 years, 39% women) (Table 1). Among preDM individuals identified bycriteria of IFG, Hemoglobin A1c (HbA1c), or impaired glucose tolerance (IGT) (n = 1433), 28% progressed to diabetes, 22% remained consistent preDM, and 50% regressed to normoglycemia. Among preDM individuals who were identified by meeting all three glycemic markers (IFG and HbA1c and IGT) (n = 200), 56% progressed to diabetes, 11% remained consistent with preDM, and 33% regressed to normoglycemia (Supplemental Table 2).

**Table 1:** Characteristics of prediabetes and euglycemic participants.

	Prediabetes (preDM)^1^				Consistent Euglycemia^1^
	Total preDM N = 974				N = 2052
		Progress n = 328 (34%)	Consistent preDM n = 272 (28%)	Regress n = 374 (38%)	
Age, mean, SD	42.8, 7.4	39.1, 7.2	46.3, 5.5	43.6, 7.6	31.6, 3.6
Women	384, 39.4%	129, 39.3%	109, 40.1%	146, 39.0%	1287, 62.7%
Black	462, 47.4%	112, 34.2%	158, 58.1%	192, 51.3%	1134, 55.3%
LE8 mean score, SD					
Total	62.11, 13.85	56.73, 12.79	64.6, 13.36	65.01, 13.74	77.69, 12.18
Diet^2^	33.57, 29.71	31.54, 29.77	37.47, 30.91	33.38, 28.78	43.01, 31.7
Physical Activity (PA)	74.1, 30.85	67.05, 32.33	78.64, 29.2	76.91, 29.68	76.26, 29.11
Nicotine exposure	64.49, 43.81	61.56, 45.79	67.78, 41.33	64.78, 43.69	63.35, 44.62
Sleep^3^	74.32, 26.74	68.55, 28.52	79.53, 23.61	77.54, 25.37	80.11, 24.77
Body mass index (BMI)	48.57, 33.34	36.9, 30.57	48.93, 32.37	58.48, 33.16	79.89, 28.33
Lipids	65.3, 29.74	63.78, 30.13	66.03, 28.78	66.1, 30.11	82.96, 24.8
Glucose	60, 0	60, 0	60, 0	60, 0	100, 0
Blood pressure (BP)	69.68, 31.97	62.24, 33.67	71.69, 31.42	74.75, 29.64	92.82, 19.09

^1^ PreDM and euglycemic status were defined by fasting glucose (FG) (FG of 100 mg/dL-125 mg/dL and FG of < 100 mg/dL respectively). Other glycemic status change categories include 1) consistent preDM (preDM throughout follow-up); 2) regression (reverted to normoglycemia during follow-up); 3) euglycemia (normoglycemia throughout). Estimates for preDM subgroups were based on data from Year 7 or following visits where the preDM was first identified. Estimates for the consistent euglycemia group were based on Year 7 data.

^2^ Diet information was only available at Y7 and Y20.

^3^ Sleep information was only available at Y15 and Y20.

**Fig. 1:**
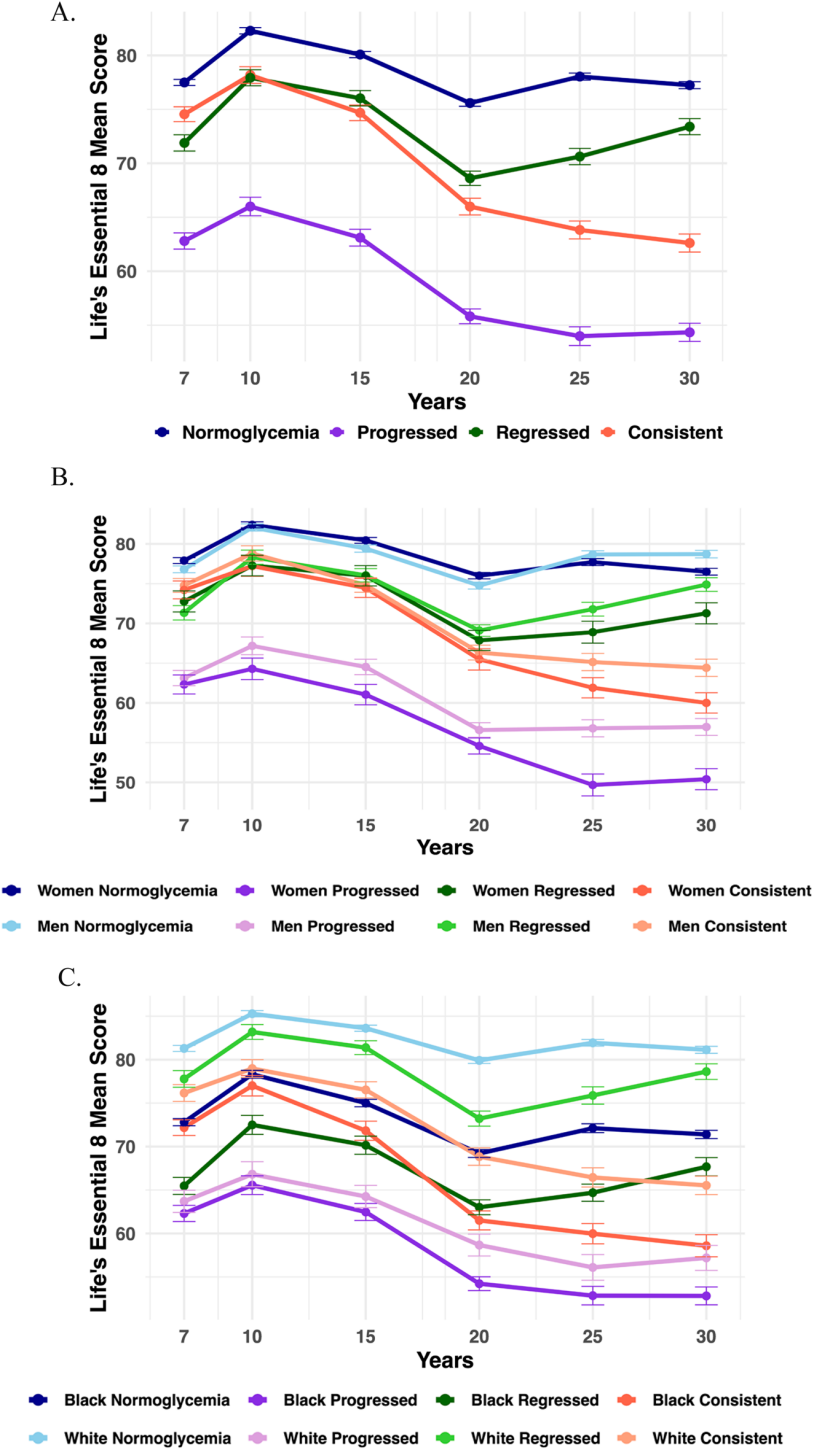
LE8 total score across years stratified by glycemic change groups, sex and race. (**A**) LE8 total score by glycemic change groups. (**B**) LE8 total score by glycemic change groups and sex. (**C**) LE8 Total score by glycemic change groups and race.

The progression group had the lowest mean LE8 total score at the time of preDM identification (57 vs. 65 in consistent preDM, 65 in regression, respectively) (Table 1) and consistently lower LE8 total score across years than the other two groups (Fig. 1A). When stratified by sex and race, we found that women and Black preDM individuals who progressed to diabetes had the lowest LE8 total scores across years than their male and White counterparts in the progression group or individuals in other glycemic change groups (Fig. 1B and C).

**Fig. 2:**
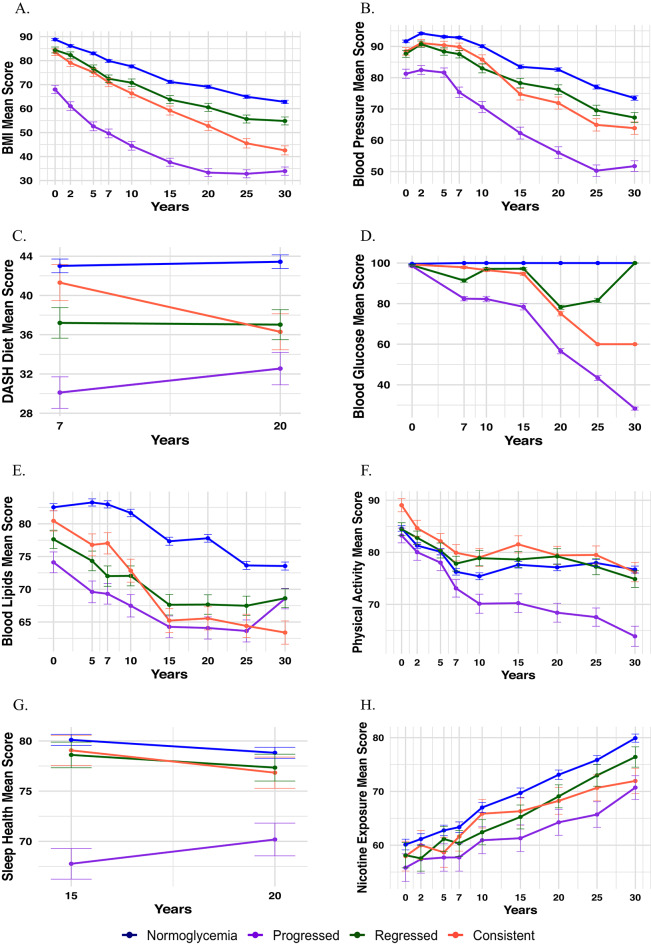
Individual scores by glycemic change groups. (**A**) BMI; (**B**) Blood Pressure; (**C**) DASH Diet; (**D**) Blood Glucose; (**E**) Blood Lipids; (**F**) Physical Activity; (**G**) Sleep Health; (**H**) Nicotine Exposure.

All LE8 component scores were the lowest in the progression group at the time of preDM identification (Fig. 2A–H). Overall, BMI, BP, lipid, glucose, and PA scores declined over time across all glycemic change groups, whereas nicotine exposure scores increased.

When stratified by sex and race (Supplemental Figs. 2 and 3), women and Black individuals in the progression group exhibited the lowest BMI and PA scores, with the steepest declines over time. Men and Black individuals in the progression group had the greatest decline in BP scores and the lowest diet scores over time. Interestingly, women and Black individuals who progressed to diabetes had the lowest sleep scores in Year 15 but improved sleep scores at Year 20 compared to other subgroups.

**Fig. 3:**
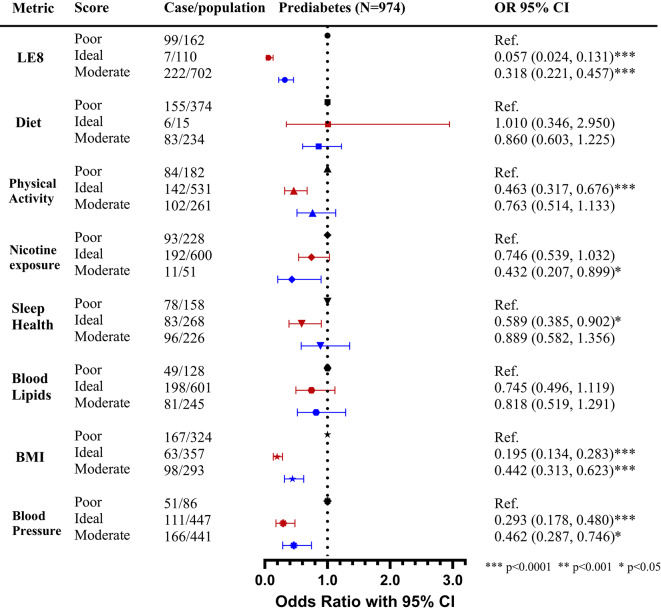
Odds ratios of Life’s Essential 8 and risk of diabetes progression among young adults with prediabetes.

Our logistic regression analysis (Fig. 3) showed that preDM participants with ideal LE8 scores had significantly lower risk of diabetes progression compared to those with poor scores (odds ratio [OR] 0.06, 95% CI 0.02–0.13, *P* < 0.0001), similarly even those participants with moderate scores as compared to poor also had reduced risk (OR 0.32, 95% CI 0.22–0.46, *P* < 0.0001). Among the individual LE8 components, BMI showed the strongest associations with reduced diabetes progression risk (Ideal BMI vs. poor BMI OR 0.2, 95% CI 0.13–0.28, *P* < 0.0001). Ideal or moderate (vs. poor) BP, PA, and sleep also demonstrated protective effects (ORs range 0.29 to 0.59, *P* < 0.05).

## Discussion

Our study highlights the dynamic nature of preDM in a young adult cohort. We demonstrated that both progression and regression are influenced by modifiable lifestyle and clinical factors, as reflected in LE8 scores. PreDM is not necessarily a permanent state, and targeted lifestyle interventions can facilitate reversion to normoglycemia^5,6^. Importantly, our study reinforces the protective role of optimal CVH metrics, as individuals with higher LE8 scores were more likely to maintain normoglycemia or regress from preDM. This is particularly relevant given long-term evidence from the UK Prospective Diabetes Study, which demonstrated that younger individuals diagnosed with type 2 diabetes (< 40 years) face a significantly higher risk of mortality compared to the general population^7^. Additionally, even after a diagnosis of diabetes, improvements in glycemic control, BP, lipid levels, and BMI have been associated with significant gains in life expectancy, underscoring the importance of early and sustained cardiovascular health optimization^8^.

Our findings further highlight the pivotal role of body weight in metabolic health; notably, individuals who remained in a preDM state or progressed to diabetes had poorer BMI scores compared to the consistently euglycemic group, suggesting that even modest weight gain over time may contribute to worsening metabolic risk. This aligns with longitudinal studies demonstrating that excess adiposity exacerbates insulin resistance and β-cell dysfunction, accelerating the transition from preDM to overt diabetes^9,10^. Lifestyle modifications have demonstrated long-term efficacy in preventing diabetes, with even a 5–10% reduction in body weight yielding substantial improvements in glucose regulation and cardiovascular outcomes ^11–14^. These findings highlight the need for proactive, targeted interventions to mitigate obesity-related metabolic risk and prevent diabetes progression.

The observed racial and sex differences in CVH metrics trends highlight critical disparities in cardiometabolic health. In our study, Black and female young adults in the progression group had the most decline in LE8 scores compared to other subgroups. This is consistent with prior evidence that demonstrated that Black adults, particularly Black women, face a disproportionately higher risk of diabetes progression due to both biological factors and social determinants^15^. Another CARDIA study analysis showed that the heightened diabetes risk in Black females was primarily driven by biological factors, with additional influence from social determinants^16^. Further research is needed to better understand the intersection of sex and racial differences in diabetes progression risk and intervention effects.

In our study, 28–56% of individuals with preDM progressed to diabetes, depending on the combination of glycemic markers for preDM definition, over 13 years of follow-up, corresponding to an annualized progression rate of 2.2–4.4%. This is lower than the percentage reported in the Diabetes Prevention Program (DPP)^5^ and the Multi-Ethnic Study of Atherosclerosis (MESA)^17^, likely reflecting differences in baseline age (preDM individuals in CARDIA mean age ~ 43 years, DPP ~ 51 years, and MESA ~ 62 years), lower baseline metabolic risk, and study design. Our study shows a critical window in early adulthood where prioritizing CVH metrics could significantly prevent or delay the onset of dysglycemia and diabetes. By addressing these modifiable risk factors during this period, there may be potential to alter long-term disease trajectories and reduce the burden of diabetes later in life.

### Limitations

Our study is among the first to estimate the CVH metrics trajectories and relationship to diabetes progression among a bi-racial cohort of young adults with preDM. However, some limitations are worth mentioning. First, the LE8 lifestyle factors were based on self-reported data. However, the instruments for these data collections were reliable and showed consistency over time^18^. Second, dietary and sleep data were only available in two CARDIA cycles, limiting the robustness of the observed secular trends. Third, HbA1c levels are known to differ by race, with African American individuals often exhibiting higher values independent of mean glucose. Our study did not adjust for this variation, consistent with prior epidemiologic studies used for comparison. As such, HbA1c values may overestimate glycemic burden in certain populations, and this should be considered when interpreting our findings. Fourth, we were unable to comprehensively assess other medications (i.e.. blood pressure and cholesterol medication) use over the long follow-up period. As the cohort aged, increased use of these medications is expected and may have influenced both glucose trajectories and the onset of diabetes, given the known metabolic effects of some antihypertensive and lipid-lowering agents. Lastly, while diverse, the study primarily includes Black and White participants, reducing its applicability to other groups.

In conclusion, our findings reinforce the importance of maintaining cardiovascular health metrics in diabetes prevention, particularly weight management, glucose control, and optimal blood pressure.

## Research design and methods

The Coronary Artery Risk Development in Young Adults (CARDIA) is an ongoing longitudinal study that examines the CVD risk factors among 5116 black and white young adults aged 18–30 years between 1985 and 1986 from four sites, including Birmingham, AL; Chicago, IL, Minneapolis, MN; and Oakland, CA. Contact is maintained with participants via telephone, mail, or email every 6 months, with annual interim medical history ascertainment. All participants provided written informed consent, with institutional review board approval at each field center (University of Alabama at Birmingham, Northwestern University, University of Minnesota, and Kaiser Permanente)^19^. We obtained data from the CARDIA study through the National Heart, Lung, and Blood Institute Biologic Specimen and Data Repository Information Coordinating Center (BioLINCC) repository, which provides access to publicly available datasets upon approval. All data were de-identified for secondary use, and no additional IRB approval was required. Our local Human Research Protection Office has deemed this analytical project as ‘Not Human Subjects Research’. We included data from Year 7 to Year 30, where glycemic and LE8 measures were consistently available. (Supplemental Table 1).

### Measurements

*PreDM* was primarily defined by impaired fasting glucose (IFG; fasting glucose (FG)100 mg/dL-125 mg/dL available in all Years) among those without a diabetes diagnosis since Year 7. We also defined preDM based on combinations of FG, hemoglobin A1c [HbA1c 5.7–6.4%; available in Years 20 and 25], and/or impaired glucose tolerance [IGT; available in Years 10, 20, and 25] (Supplemental Table 2).

*The risk of diabetes progression* was defined by FG ≥ 126 mg/dL (and/or diagnostic level of HbA1c and oral glucose tolerance) or a diabetes diagnosis in the following visits among those preDM.

Other glycemic status change categories include 1) consistent preDM (preDM throughout follow-up); 2) regression (reverted to normoglycemia during follow-up); 3) euglycemia (normoglycemia throughout).

*LE8 scores* include diet, physical activity (PA), nicotine exposure, sleep health, BMI, blood lipids, blood glucose, and blood pressure (BP). Each component was scored 0–100, creating a composite CVH score per AHA guidelines^3^. We further categorized the overall and component scores as ideal, moderate, or poor (Supplemental Table 1).

### Statistical analyses

We estimated LE8 overall and component mean scores across glycemic change categories, adjusted for age, race, and sex from Year 7 to Year 30. We performed logistic regression to assess the association between LE8 scores and the risk of diabetes progression, adjusted for age at preDM identification, race, and sex.

## Supplementary Information

Supplementary Information.

## Data Availability

Data used for this study were obtained from the National Heart, Lung, and Blood Institute Biologic Specimen and Data Repository Information Coordinating Center (BioLINCC) which provides access to publicly available datasets upon approval at https://biolincc.nhlbi.nih.gov/studies/cardia/. Our approved access ID is 13815. Tulane Human Research Protection Office has deemed this analytical project as ‘Not Human Subjects Research’ September 15, 2023, Reference# 2023–1373. The study data inquiries should be addressed to the corresponding author (Yilin Yoshida, PhD., yyoshida1@tulane.edu).
