# Supplementary material for: Life’s essential 8 and risk of progression to diabetes among young adults with prediabetes
Source: Sci Rep. 2025 Oct 13;15:35690. doi: 10.1038/s41598-025-19472-y (PMC12518763; doi:10.1038/s41598-025-19472-y)
Supplement: Supplementary file 1 — Supplementary Information. [file 41598_2025_19472_MOESM1_ESM.pdf]

Supplemental Table 1. Life's Essential 8 Definitions

Variables	LE8 Score	Category	Definition
Diet [1](self-reported daily intake of a DASH-style eating pattern)	80-100<br>50-80<br>0-50	Ideal<br>Intermediate<br>Poor	75th-95th percentile<br>50th-75th percentile<br>1st-50th percentile
Physical activity (self-reported minutes of moderate or vigorous PA per week)	80-100<br>60-80<br>0-60	Ideal<br>Intermediate<br>Poor	90-150 min/week<br>60-90 min/week<br>0-60 min/week
Tobacco/nicotine exposure (self-reported use of cigarettes or inhaled NDS)	75-100<br>25-75<br>0-25	Ideal<br>Intermediate<br>Poor	Never smoker<br>Former smoker<br>Current smoker
Sleep (self-reported average hours of sleep per night)	90-100<br>70-90<br>0-70	Ideal<br>Intermediate<br>Poor	7-9 h/night<br>6-7 h/night<br><6 or >10 h/night
BMI (body weight [kg] divided by height squared [meters squared])	70-100<br>30-70<br>0-30	Ideal<br>Intermediate<br>Poor	<29.9 kg/m2<br>30-35 kg/m2<br>35-40+ kg/m2
Lipids (plasma total and HDL cholesterol with calculation of non-HDL cholesterol)	60-100<br>40-60<br>0-40	Ideal<br>Intermediate<br>Poor	<159 mg/dL<br>160-189 mg/dL<br>190-200þmg/dL
Glucose (FG)	60-100<br>40-60<br>0-40	Ideal<br>Intermediate<br>Poor	Non-diabetic<br>DM, FG<126 mg/dL<br>DM, FG>=126 mg/dL
Blood pressure (appropriately measured systolic and diastolic BPs)	75-100<br>25-75<br>0-25	Ideal<br>Intermediate<br>Poor	<129/<80 mmHg<br>Stage 1 hypertension<br>>140/>90 mmHg

1 Diet information was only available at Y7 and Y20. We generated a DASH score based on prior work by Mellen et al., which is also one of the original diet metrics methods cited by the AHA. [1] Briefly, we identified DASH goals for 8 target nutrients (total fat, saturated fat, protein, fiber, cholesterol, calcium, magnesium, and potassium) and the DASH score was generated by the sum of all nutrient targets met (maximum score, 9). Individuals with intake meeting a goal intermediate between the DASH goal and the nutrient content of the DASH control diet were given a score of 0.5 for that nutrient.[1]

2 Physical activity was assessed with an interviewer-administered self-report of leisure-time frequency and duration of participation in 13 specific activities over the past 12 months. [2]

References

1. Mellen PB, Gao SK, Vitolins MZ, Goff DC, Jr. Deteriorating dietary habits among adults with hypertension: DASH dietary accordance, NHANES 1988-1994 and 1999-2004. *Arch Intern Med*. Feb 11 2008;168(3):308-14. doi:10.1001/archinternmed.2007.119
2. Sidney S, Jacobs DR, Jr., Haskell WL, et al. Comparison of Two Methods of Assessing Physical Activity in the Coronary Artery Risk Development in Young Adults (CARDIA) Study. *American Journal of Epidemiology*. 1991;133(12):1231-1245. doi:10.1093/oxfordjournals.aje.a115835

Supplemental Table 2. Proportions of Glycemic Change Groups According to Different Prediabetes Definitions

	**Available Years**	**Mean follow-up Years**	**Progress**	**Consistent**	**Regress**
IFG **(primary)**<br>N=974	All (Y7 to Y30)	12.7, 6.8	328, 33.7%	272, 27.9%	374, 38.4%
IFG **or** HbA1c 5.7%-6.4% **or** IGT<br>N=1433	HbA1c (Y20 and Y25)<br><br>OGTT (Y10, Y20, and Y25)	12.8, 6.1	405, 27.9%	323, 22.3%	723, 49.8%
IFG **and** HbA1c 5.7%-6.4% **and** IGT<br>N=200		12.7, 6.0	74, 56.1%	15, 11.4%	43, 32.6%

We used fasting glucose as our primary marker to define glycemic change status because it is consistently available from Y7 to Y30.

IFG: impaired fasting glucose; HbA1c: Hemoglobin A1c; IGT: impaired glucose tolerance

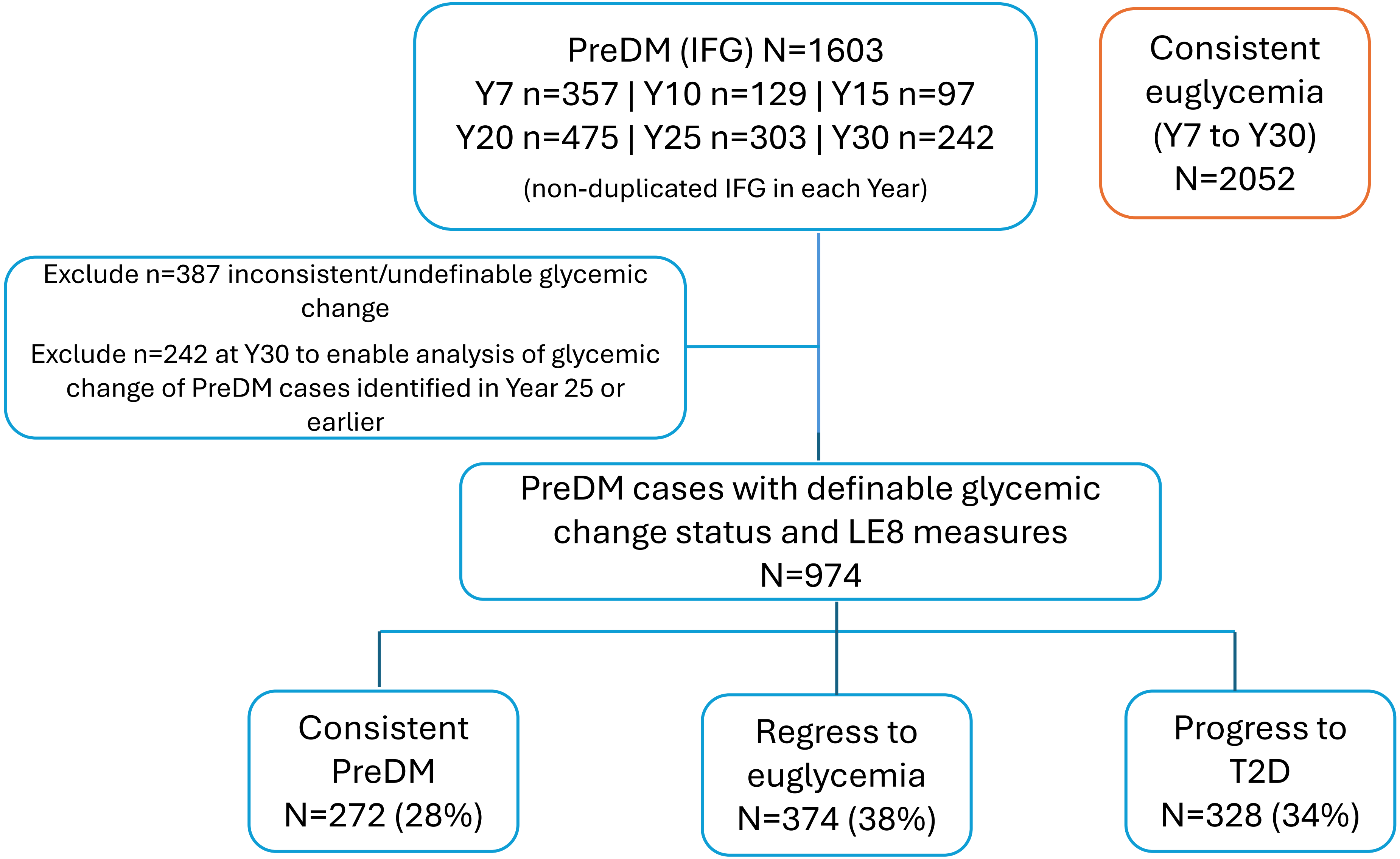

Supplemental Figure 1. Sample Selection

Supplemental Figure 2.A. BMI

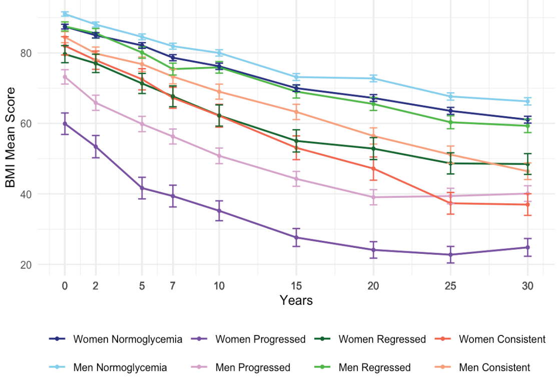

Supplemental Figure 2.B. Blood Pressure

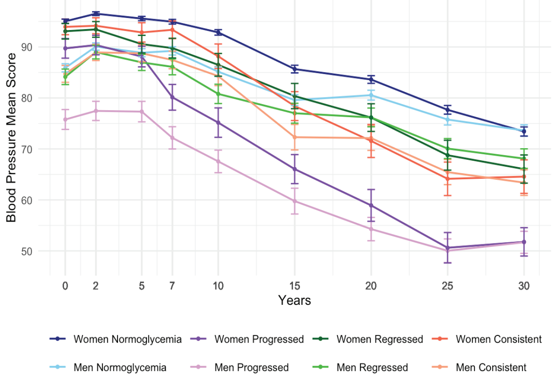

Supplemental Figure 2.C. DASH Diet

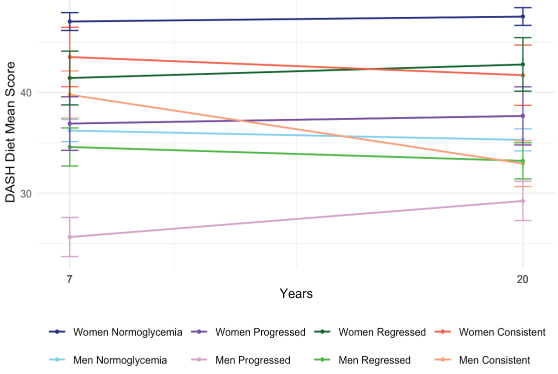

Supplemental Figure 2.D. Blood Glucose

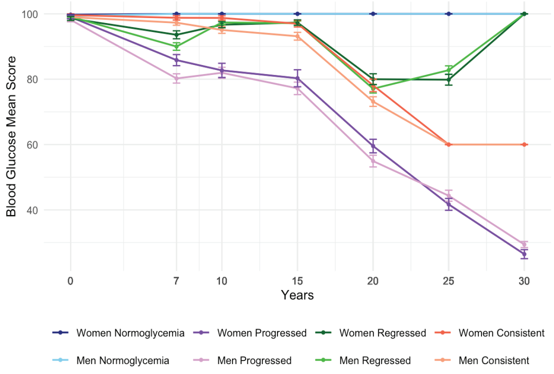

Supplemental Figure 2.E. Blood Lipids

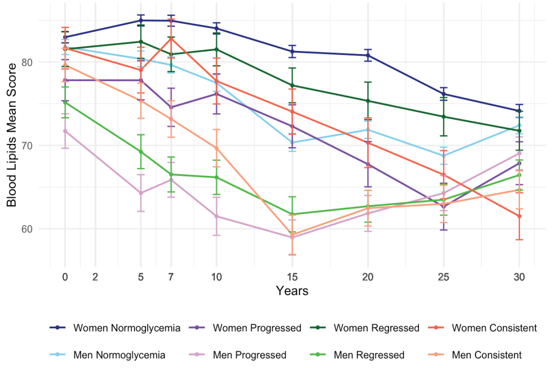

Supplemental Figure 2.F. Physical Activity

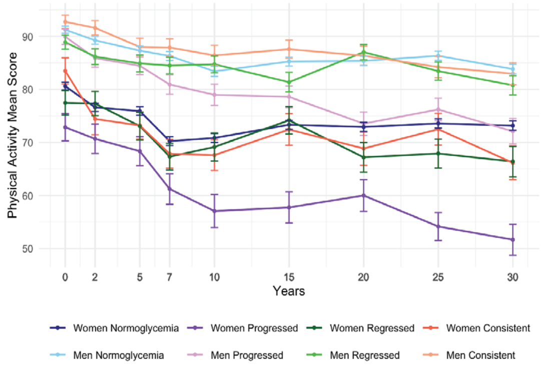

Supplemental Figure 2.G. Sleep Health

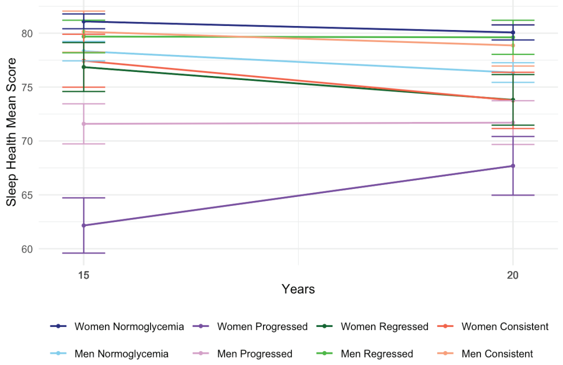

Supplemental Figure 2.H. Nicotine Exposure

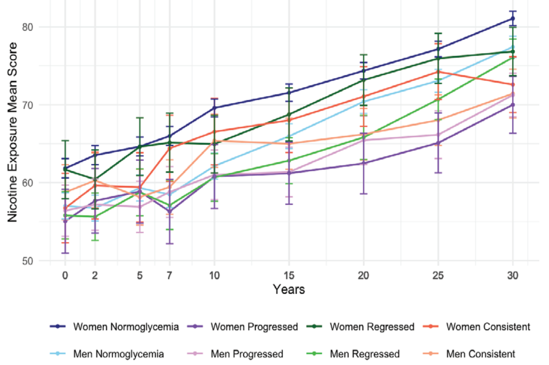

Supplemental Figure 2. LE8 Component Scores by Glycemic Change Groups and Sex

Supplemental Figure 3.A. BMI

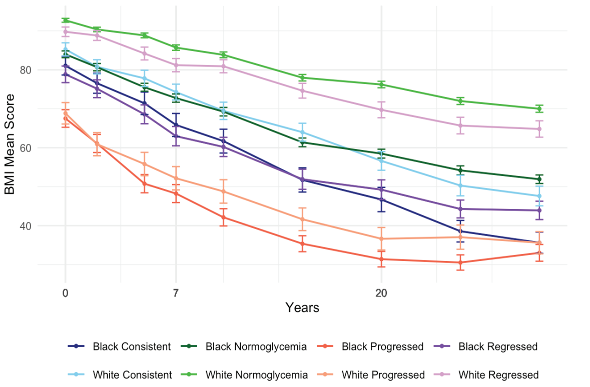

Supplemental Figure 3.B. Blood Pressure

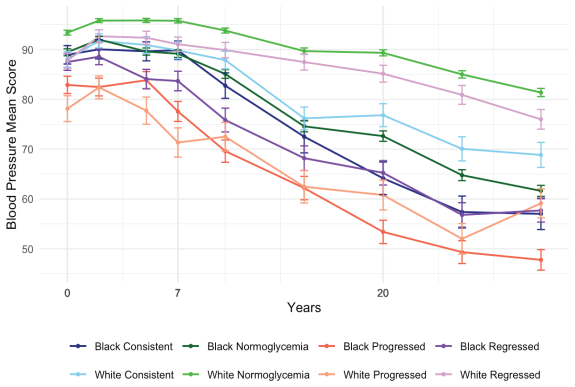

Supplemental Figure 3.C. DASH Diet

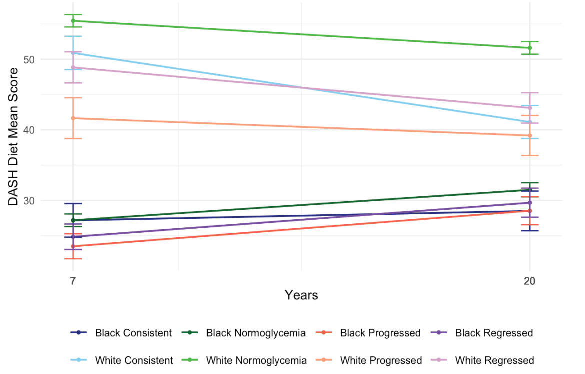

Supplemental Figure 3.D. Blood Glucose

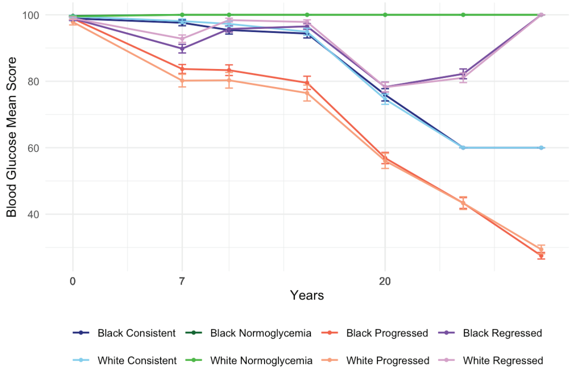

Supplemental Figure 3.E. Blood Lipids

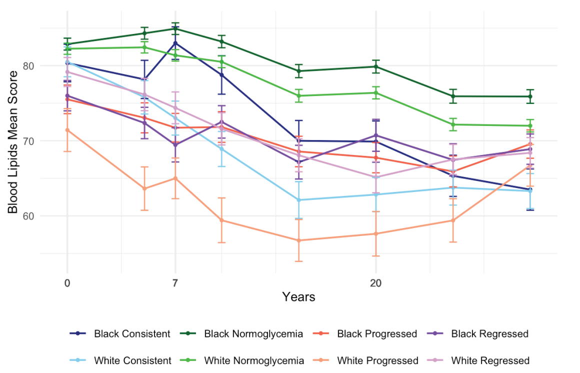

Supplemental Figure 3.F. Physical Activity

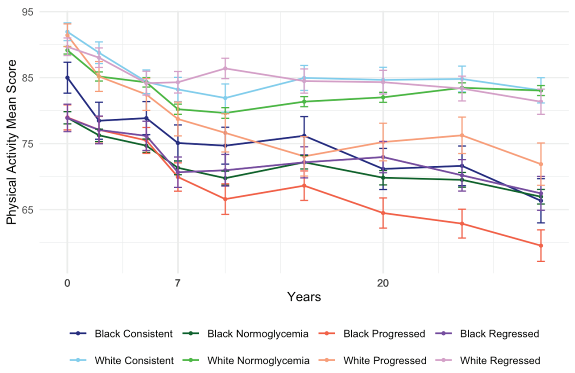

Supplemental Figure 3.G. Sleep Health

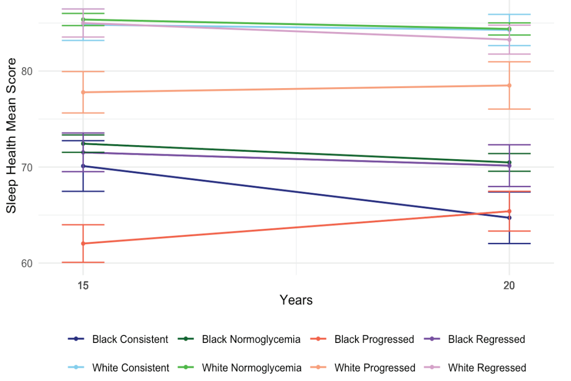

Supplemental Figure 3.H. Nicotine Exposure

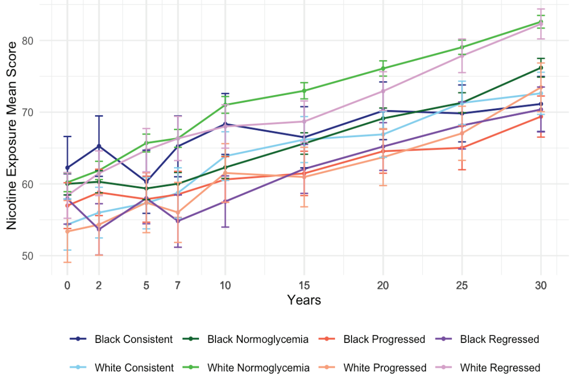

Supplemental Figure 3. LE8 Component Scores by Glycemic Change Groups and Race
